# A Lys49 Phospholipase A_2_, Isolated from *Bothrops asper* Snake Venom, Induces Lipid Droplet Formation in Macrophages Which Depends on Distinct Signaling Pathways and the C-Terminal Region

**DOI:** 10.1155/2013/807982

**Published:** 2012-12-24

**Authors:** Karina Cristina Giannotti, Elbio Leiguez, Vanessa Moreira, Neide Galvão Nascimento, Bruno Lomonte, José Maria Gutiérrez, Robson Lopes de Melo, Catarina Teixeira

**Affiliations:** ^1^Laboratory of Pharmacology, Butantan Institute, Avenida Vital Brazil, 05503-900 São Paulo, SP, Brazil; ^2^Clodomiro Picado Institute, School of Microbiology, University of Costa Rica, 2060 San José, Costa Rica; ^3^Center for Applied Toxinology (CAT), Butantan Institute, 05503-900 São Paulo, SP, Brazil

## Abstract

MT-II, a Lys49PLA_2_ homologue devoid of catalytic activity from *B. asper* venom, stimulates inflammatory events in macrophages. We investigated the ability of MT-II to induce formation of lipid droplets (LDs), key elements of inflammatory responses, in isolated macrophages and participation of protein kinases and intracellular PLA_2_s in this effect. Influence of MT-II on PLIN2 recruitment and expression was assessed, and the effects of some synthetic peptides on LD formation were further evaluated. At noncytotoxic concentrations, MT-II directly activated macrophages to form LDs. This effect was reproduced by a synthetic peptide corresponding to the C-terminal sequence 115–129 of MT-II, evidencing the critical role of C-terminus for MT-II-induced effect. Moreover, MT-II induced expression and recruitment of PLIN2. Pharmacological interventions with specific inhibitors showed that PKC, PI3K, ERK1/2, and iPLA_2_, but not P38^MAPK^ or cPLA_2_, signaling pathways are involved in LD formation induced by MT-II. This sPLA_2_ homologue also induced synthesis of PGE_2_ that colocalized to LDs. In conclusion, MT-II is able to induce formation of LDs committed to PGE_2_ formation in a process dependent on C-terminal loop engagement and regulated by distinct protein kinases and iPLA_2_. LDs may constitute an important inflammatory mechanism triggered by MT-II in macrophages.

## 1. Introduction

Phospholipases A_2_s (PLA2; EC 3.1.1.4) constitute a family of lipolytic enzymes with key roles in several cellular processes by regulating the release of arachidonic acid and lysophospholipids from cell membrane phospholipids. Venoms from snakes of the Viperidae family contain group IIA phospholipases A_2_ (PLA_2_s), which share structural and functional features with PLA_2_s found in inflammatory exudates in mammals [1, 2]. A number of *Bothrops* snake venom PLA_2_s have been shown to induce inflammatory events such as edema and leukocyte infiltration and to directly activate inflammatory cell functions [3–6]. 

Basic PLA_2_s are considered the most important venom components responsible for the severe local myotoxicity and inflammation characteristic of the envenomation induced by* Bothrops* genus snakes [7]. These enzymes are further divided into two subgroups, namely, catalytically active variants, presenting a conserved aspartic acid residue at position 49 (Asp49PLA_2_s), and catalytically inactive homologues, known as Lys49PLA_2_s, which present various substitutions in residues of the Ca^2+^ binding loop, as well as at position 49, where Lys replaces the highly conserved Asp [8, 9]. Such modifications drastically affect the catalytic ability of these proteins rendering these homologues enzymatically inactive [10]. Interestingly, Lys49PLA_2_ homologues are highly myotoxic, bactericidal, and proinflammatory [9], evidencing that phospholipid hydrolysis is not strictly required for these activities. Studies on synthetic peptides and site-directed mutagenesis identified the C-terminal region of Lys49PLA_2_s as essential for their biological activities [10, 11]. Thus, Lys49PLA_2_ homologues constitute interesting models to investigate a series of cellular effects which do not depend on membrane phospholipid hydrolysis. 

In the *Bothrops asper* snake venom three myotoxic Lys49-PLA_2_s have been identified, named MT-II, MT-IV, and M1-3-3, and reported in UNIPROT database. Besides myotoxicity, MT-II, the most studied Lys49PLA_2_ homologue, has been reported to induce inflammation *in vivo* [5, 12] and to activate some inflammatory functions of macrophages *in vitro*, increasing phagocytosis, respiratory burst, and release inflammatory mediators [4] at noncytotoxic concentrations. However, the knowledge on the effects of this Lys49PLA_2_ in macrophages functions, is still fragmentary.

Macrophages play key roles in a wide variety of processes associated with tissue maintenance, antigen presentation, inflammation, and tissue repair [13]. Upon inflammatory stimuli, quiescent macrophages become activated and present increased number of lipid rich cytoplasmic organelles named lipid droplets (LDs), also known as lipid bodies. These organelles are functionally involved in biosynthesis, transport, and catabolism of lipids [14, 15] as well as biosynthesis and accumulation of inflammatory mediators, such as eicosanoids and cytokines [16, 17]. Moreover, leukocyte LDs associated with inflammatory responses have been shown to compartmentalize signaling proteins involved in cellular activation and structural proteins, mainly perilipin 2 (PLIN2), also named adipophilin (Adipose differentiation-related protein, ADRP), which has an important role in LD assembly and formation of foam macrophages [18, 19], which are markers of atherosclerotic plaques [19]. Increased numbers of lipid droplets are described in distinct populations of leukocytes during inflammatory and infectious processes [20, 21]. Recently, MT-III, a catalytically active variant Asp49PLA_2_ from *B. asper* venom, has been shown to activate macrophages to form increased amounts of LDs [22], but no such effect has been described for the action of Lys49PLA_2_s. Therefore, it is relevant to assess the effects of MT-II on macrophages in terms of LD formation. Such macrophage activation might play a relevant role in the scenario of the local pathological alterations induced by snake venom toxins. Based on these information, in the present study the ability of MT-II to induce LD formation in macrophages was evaluated and the mechanisms involved in this effect were analyzed in terms of recruitment and expression of PLIN2, participation of intracellular PLA_2_s (cPLA_2_ and iPLA_2_) and signaling protein kinases. In light of the absence of catalytic activity in MT-II, the effects of some synthetic peptides related to distinct regions of this Lys49PLA_2_ molecule on lipid droplet formation were further evaluated in macrophages. 

## 2. Materials and Methods 

### 2.1. Chemicals and Reagents

MTT and L-glutamine were obtained from USB Corporation (Cleveland, OH, USA). H7, LY294002, SB202190, PD98059, and Pyr-2 were purchased from Calbiochem-Novabiochem (La Jolla, CA, USA). Racemic mixture of BEL and anti-mouse PGE_2_ was obtained from Cayman Chemical (Ann Arbor, MI, USA). Guinea pig polyclonal antibody anti-mouse PLIN2 and FITC-conjugated donkey anti-guinea pig antibody were obtained from Research Diagnostics Inc. (Flanders, NJ, USA). Secondary antibodies anti-mouse and anti-guinea pig conjugated to horseradish peroxidase and nitrocellulose membrane were obtained from GE Healthcare (Buckinghamshire, UK). Gentamicin was purchased from Schering-Plough, NJ, USA). DMSO and BSA were obtained from Amresco (Solon, OH, USA). Mouse monoclonal antibody anti-*β*-actin, Nile Red, RPMI-1640, thiocarbohydrazide, OsO_4_, and EDAC were purchased from Sigma Aldrich Co. (St. Louis, MO, USA). PFA was purchased from Electron Microscopy Science (USA). Alexa Fluor 488 Goat Anti-mouse IgG was purchased from Life Technologies (Grand Island, NY, USA). DAPI and fluoromount G were purchased from Molecular Probes (Eugene, OR, USA). Donkey serum was obtained from Jackson ImmunoResearch Laboratories (PA, USA). Triton-X was obtained from Union Carbide Corporation (Danbury, USA). GA, thioglycolate, and all salts used were obtained from Merck (Darmstadt, Germany). 

### 2.2. Animals

Male Swiss mice (18–20 g) were obtained from Butantan Institute (São Paulo, Brazil). Animals were housed in a temperature-controlled room (22–24°C) with a 12 h light-dark cycle and fresh water and food *ad libitum* until used. This study was approved by the Butantan Institute Animal Experimentation Ethics Committee (reference number 760/10) in accordance with the procedures laid down by the Universities Federation for Animal Welfare. 

### 2.3. Phospholipase A_2_

The Lys49PLA_2_ homologue (MT-II) was isolated from *Bothrops asper* venom by ion-exchange chromatography on CM-Sephadex C-25 as described [23], followed by RP-HPLC on a C8 semipreparative column (10 × 250 mm; Vydac) eluted at 2.0 mL/min with a 0–70% acetonitrile gradient containing 0.1% trifluoroacetic acid, during 30 min, on an Agilent 1200 instrument monitored at 215 nm. Homogeneity was assessed by analytical reverse-phase HPLC on a C4 column using a gradient of 0–60% acetonitrile in 0.1% trifluoroacetic acid (v/v). The absence of endotoxin contamination in the MT-II preparation was demonstrated by the quantitative *Limulus* amebocyte lysate (LAL) test [24], which revealed undetectable levels of endotoxin (<0.125 EU/mL).

### 2.4. Synthetic Peptides

The following synthetic peptides were synthesized and used for biological assays: (a) peptide 115–129 (KKYRYYLKPLCKK) corresponding to the original sequence 115–129 of MT-II from *B. asper* snake venom; (b) peptide p115-W3 (KKWRWWLKPLCKK) corresponding to the triple tyrosine-to-tryptophan substitution of p115-129; (c) peptide pEM-2 (KKWRWWLKALAKK), in which the proline and cysteine residues of p115-W3 were each replaced by an alanine residue; (d) peptide 60–71 (KKDRYSYSWKDK) corresponding to a central region sequence of MT-II; (e) peptide p-Scr (FKFKYKKACKKYK) corresponding to a scrambled peptide version of the sequence 115–129 of ACL myotoxin, a Lys49 PLA_2_ homologue from the venom of the snake *Agkistrodon contortrix laticinctus*.

Synthetic peptides were prepared in automated bench-top simultaneous multiple solid-phase synthesizer (PSSM 8 system from Shimadzu Co.) using solid-phase peptides synthesis by the Fmoc procedure [25]. Briefly, sequential couplings of protected amino acids were performed with HOBt, TBTU and NMM on Fmoc-Lys(Boc)-Wang resin (Merck KGaA, Germany). Fmoc group cleavage was performed with 30% piperidine (v/v) in DMF. The resin-bound peptides were cleaved/deprotected with TFA/thioanisole/EDT/phenol/water (82.5 : 5: 2.5 : 5 v/v/v/v) at room temperature for 4 h. After filtration, the filtrate was concentrated under argon stream and precipitated with diethyl ether. All crude peptides were purified by reversed-phase chromatography (Shim-pack Prep-ODS, Shimadzu Co.) semipreparative HPLC, and the purity and identity of the peptide were confirmed by mass spectrometry and by analytical HPLC. 

### 2.5. Harvesting of Macrophages

Peritoneal macrophages were harvested 4 days after i.p. injection of 1 mL of 3% thioglycolate. Animals were killed under CO_2_ atmosphere and cells were harvested by washing peritoneal cavities with 3 mL of PBS, pH 7.2, containing 10 IU/mL heparin. Aliquots of the washes were used for total cell counts in a Neubauer chamber after dilution (1 : 20, v/v) in Turk solution (0.2% crystal violet dye in 30% acetic acid). Differential cell counts were performed on smears stained with Hema3. More than 95% of the cell population consisted of macrophages, as determined by conventional morphological criteria. The remaining wash volumes were centrifuged at 500 ×g for 6 min (4°C) and the cell pellets were used for subsequent studies after suitable dilutions.

### 2.6. Cytotoxicity Assay

Cytotoxicity of MT-II towards elicited macrophages was evaluated using the MTT assay. In brief, 2 × 10^5^ macrophages/well in RPMI-1640 medium supplemented with 40 *μ*g/mL gentamicin sulfate and 2 mM L-glutamine were plated in 96-well plates and incubated with 100 *μ*L of selected concentrations of MT-II (0.4–0.8 *μ*M) diluted in medium or with the same volume of medium alone (control) for 1, 6, 12, and 24 h at 37°C in a humidified atmosphere (5% CO_2_). MTT (5 mg/mL) was dissolved in PBS and filtered for sterilization and removal of a small amount of insoluble residue present in some batches of MTT. Stock MTT solution (10% in culture medium) was added to all wells in each assay, and plates were incubated at 37°C for 3 h. One hundred *μ*L of DMSO were added to all wells and mixed thoroughly at room temperature for 30 min. Absorbances at 540 nm were then recorded in a microtiter plate reader. Results were expressed as percentage of viable cells, considering control cells incubated with medium alone as 100% viable. 

### 2.7. Stimulation and Treatment of Macrophages

Macrophages were plated on glass coverslips in 24-well plates at a density of 2 × 10^5^ cells/coverslip and allowed to attach for 30 min at 37°C under a 5% CO_2_ atmosphere. Non-adherent cells were removed by washing with PBS. Cell monolayers were cultured for 1 h in RPMI-1640 supplemented with 40 *μ*g/mL gentamicin sulfate and 2 mM L-glutamine at 37°C and 5% CO_2_ and were then challenged with selected concentrations of MT-II (0.2–1.2 *μ*M) or synthetic peptides (250 *μ*g/mL) or medium (control). Where appropriate, the following inhibitors were used: 1 *μ*M SB202190, inhibitor of p38MAPK; 1 *μ*M LY294002, inhibitor of PI3 K; 6 *μ*M H7-Dihydro, inhibitor of PKC; 25 *μ*M PD98059, inhibitor of ERK1/2; 1 *μ*M Pyr-2 (Pyrrolidine-2), inhibitor of cPLA_2_ and 2 *μ*M BEL (bromoenol lactone) an inhibitor of iPLA_2_. All stock solutions were prepared in DMSO and stored at −20°C. Aliquots were diluted in RPMI-1640 to the required concentration immediately before use. The final DMSO concentration was always lower than 1% and had no effect on lipid body numbers. All pharmacological inhibitors were added between 30 and 60 min before stimulation of macrophages with MT-II or medium (control). Cells treated with the inhibitors were analyzed for viability by the tetrazolium-based (MTT) colorimetric assay. No significant changes in cell viability were registered with any of the above agents or vehicle at the concentrations used (data not shown).

### 2.8. Lipid Body Staining and Quantification

Analysis of lipid body numbers was performed in osmium-stained cells. In brief, macrophages (2 × 10^5^ cells) adhered to glass coverslips were fixed in 4% PFA in 0.1 M phosphate buffer, pH 7.2, for 15 min, and stained with OsO_4_. The coverslips were then rinsed in 0.1 M phosphate buffer, stained in 1% OsO_4_ (30 min), rinsed in deionized H_2_O, immersed in 1.0% thiocarbohydrazide (5 min), rinsed again in 0.1 M phosphate buffer, restained with 1% OsO_4_ (3 min), rinsed with H_2_O, and then dried and mounted. The morphology of the fixed cells was observed and round osmiophilic structures were identified as lipid droplets, which were then counted under phase-contrast microscopy using the 100x objective lens in 50 consecutively scanned leukocytes in each coverslip. For assays with fluorescent-labeled lipid droplets, macrophages (2 × 10^5^ cells) adhered to glass coverslips were incubated with Nile Red staining solution freshly prepared in 0.1 M phosphate buffer (10 *μ*g/mL) for 20 min at room temperature and washed with phosphate buffer. After several washes the coverslips were mounted with fluoromount G and examined under a fluorescence microscope equipped with the appropriate filter (Zeiss LSM 510 Meta). 

### 2.9. Electron Microscopy

A standardized protocol for electron microscopy procedure was developed for ultrastructural analysis of lipid droplets. Macrophages (5 × 10^6^ cells) incubated with either MT-II (0.8 *μ*M) or medium alone for 1 h were fixed in a diluted mixture of freshly prepared aldehydes (4% PFA/1% GA in 0.1 M phosphate buffer) containing 3.5% sucrose for 2 h at room temperature and then washed three times in 0.1 M phosphate buffer. Cells were centrifuged at 129 ×g, and cell pellets were postfixed with OsO_4_ (1% in phosphate buffer at room temperature) followed by three washes with saline solution. Uranyl acetate in aqueous solution was then added for 2 h at room temperature before dehydration in a graded series of ethanol (70, 95 and 100% twice for 10 min each). For embedding, aliquots of propylene oxide were added twice for 10 minutes, followed by Spurr resin diluted in propylene oxide (1 : 1) and undiluted Spurr resin for 12 h. Polymerization was carried out for 48 h at 70°C. Samples were then incubated with 4% uranyl acetate and lead citrate for contrast. Thin sections were examined with LEO 906E and Zeiss EM109 transmission electron microscopes.

### 2.10. Immunodetection of PLIN2 and PGE_2_

Detection of PLIN2 in MT-II-stimulated macrophages was performed by PLIN2 immunostaining. In brief, macrophages attached to coverslips and stimulated for 3 h with MT-II (0.8 *μ*M) were fixed in 2% paraformaldehyde (PFA). The cells were permeabilized with 0.2% Triton-X 100 in 0.1 M phosphate buffer and blocked with 0.5% normal donkey serum in 0.1 M phosphate buffer for 90 min. After PBS washes, macrophages were incubated for 1 h with guinea pig polyclonal anti-mouse PLIN2 (1 : 2000) diluted in 0.1 M phosphate buffer with 0.2% Triton-X 100. After three washes with PBS (10 min each), the preparations were incubated for 1 h with secondary FITC-conjugated donkey anti-guinea pig antibody (1 : 500) in the dark for 1 h. After the washes, the slides were mounted with fluoromount G and examined under confocal laser scanning microscope (Zeiss LSM 510 Meta). For analysis of PGE_2_ immunostaining, the cell were fixed and permeabilized in 1% N-ethyl-N′-(3-dimethylaminopropyl) carbodiimide hydrochloride (EDAC) in HBSS^−^/^−^. The macrophages were blocked with 0.5% normal donkey serum in 0.1 M phosphate buffer for 60 min. Next, the macrophages were washed with HBSS^−^/^−^ and incubated for 1 h with anti-PGE_2_ (1 : 100). After further washes, cells were incubated with biotinylated rabbit anti-mouse IgG secondary Ab (1 : 250) and Nile red solution (1 : 250) in the dark for 1 h. The cover slips were then washed three times and mounted with fluoromount G containing DAPI (Vector Laboratories, Burlingame, CA) and examined under confocal laser scanning microscope (Zeiss LSM 510 Meta). 

### 2.11. Western Blotting of PLIN2

Aliquots of MT-II-stimulated and -nonstimulated cells (2 × 10^6^ cells) were lysed with 100 *μ*L of sample buffer (0.5 M Tris-HCl, pH 6.8, 20% SDS, 1% glycerol, 1 M *β*-mercaptoethanol, and 0.1% bromophenol blue) and boiled for 10 min. Samples were resolved by SDS polyacrylamide gel electrophoresis (SDS-PAGE) on 10% bis-acrylamide gels overlaid with a 5% stacking gel. Proteins were then transferred to nitrocellulose membrane (GE Healthcare, Buckinghamshire, UK) using a Mini Trans-Blot (Bio-Rad Laboratories, Richmond, CA, USA). The membranes were blocked for 1 h with 5% nonfat dry milk in TTBS (20 mM Tris, 100 mM NaCl and 0.5% Tween 20) and incubated with primary antibodies against PLIN2 (1 : 2000 dilution) and *β*-actin (1 : 3000) for 1 h. They were then washed and incubated with the appropriate secondary antibody conjugated to horseradish peroxidase. Detection was by the enhanced chemiluminescence (ECL) method according to the manufacturer's instructions (GE Healthcare, Buckinghamshire, UK). Band densities were quantified with a GS 800 Densitometer (Bio-Rad Laboratories, Richmond, CA) using the image analysis software from Molecular Analyst (Bio-Rad Laboratories, Richmond, CA, USA).

### 2.12. Statistical Analysis

Data are expressed as the mean ± standard error of mean (SEM) of at least three independent experiments. Multiple comparisons among groups were performed by one-way analysis of variance (ANOVA) followed by Tukey's test. Values of probability lower than 5% (*P* < 0.05) were considered significant.

## 3. Results

### 3.1. Effect of MT-II on Macrophage Viability

**Figure 1:**
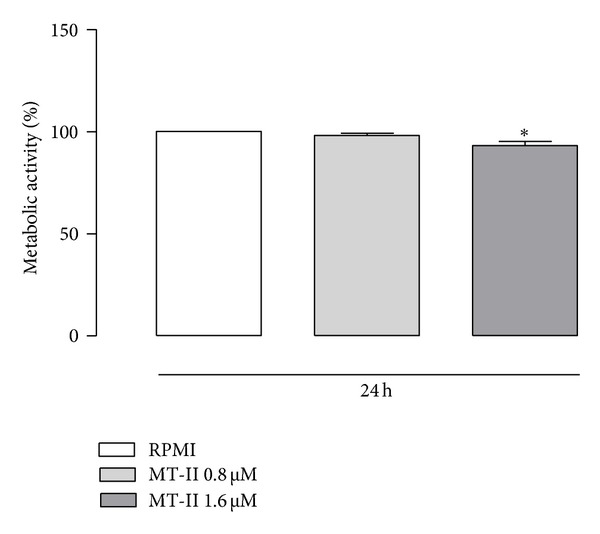
Effect of MT-II on cell viability. The cells were incubated with MT-II (0.8 and 1.6 *μ*M) or RPMI (control) for 24 h, and cytotoxicity was assessed by the tetrazolium-based (MTT) colorimetric assay. Values represent the mean ± SEM from four animals. *P < 0.05 compared with control (RPMI).

Initially, the effect of MT-II on isolated-elicited macrophage viability was assessed by the tetrazolium-based (MTT) colorimetric assay. To this purpose the effect of 24 h incubation with two distinct concentrations of MT-II (0.8 and 1.6 *μ*M) were evaluated. As shown in Figure 1, incubation of macrophages with MT-II at a concentration of 0.8 *μ*M did not affect macrophage viability. At a concentration of 1.6 *μ*M, the sPLA_2_ homologue partially decreased (*P* < 0.05) macrophage viability. 

### 3.2. MT-II Induces LD Formation in Macrophages

**Figure 2:**
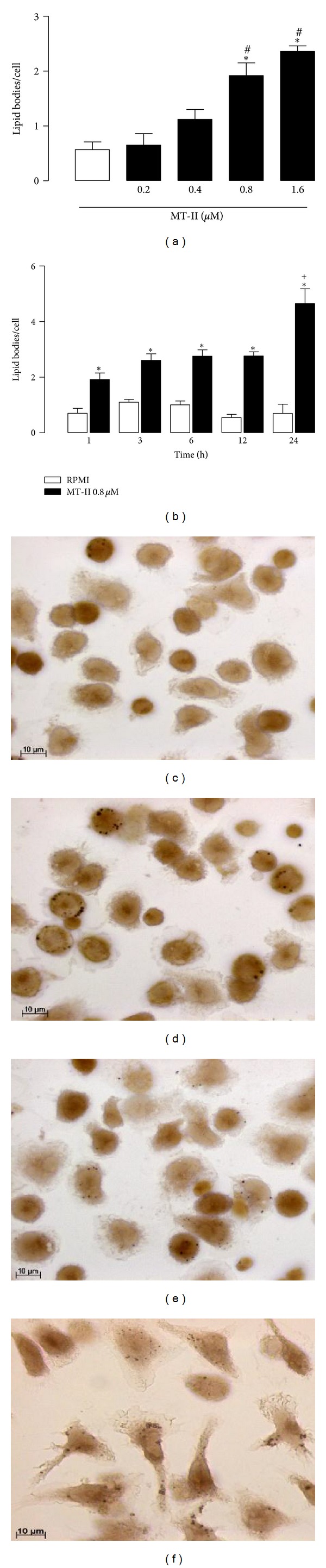
MT-II induces formation of LDs in peritoneal macrophages in culture.(a) Effect of selected concentrations of MT-II on formation of LDs in macrophages which were incubated with various concentrations of MT-II or with RPMI (Control) for 1 h. (b) Time-course of MT-II-induced LD formation. Macrophages were incubated with MT-II (0.8 *μ*M) or RPMI (Control) for 1, 3, 6, 12, or 24 h. LDs were quantified using light microscopy after osmium staining. LDs after osmium staining observed in control (c) or in cells stimulated with MT-II (0.8 *μ*M) for 1 h (d), 12 h (e), or 24 h (f). Each bar represents the mean ± S.E.M. of the number of LDs/cell in 50 counted cells. Values represent means ± S.E.M. for three to five animals. *P < 0.05 compared with control cells; ^#^ *P* < 0.05 compared with cells stimulated by 0.2 or 0.4 *μ*M of MT-II; ^+^ *P* < 0.05 compared with cells stimulated by MT-II (0.8 *μ*M) for 1, 3, 6 and 12 h.

 To determine whether stimulation of peritoneal macrophages with MT-II would lead to LDs formation, these cells were incubated with selected concentrations of MT-II (0.2–1.6 *μ*M) for 1 h. As demonstrated in Figure 2(a), incubation of macrophages with MT-II at concentrations from 0.8 to 1.6 *μ*M, but not from 0.2 and 0.4 *μ*M, for 1 h induced a significant increase (*P* < 0.05) in the number of LDs in comparison with control cells incubated with culture medium alone. Maximal LD numbers were observed at 1.6 *μ*M MT-II. To determine the time-course of LD formation induced by MT-II, a submaximal concentration of this Lys49PLA_2_ was used (0.8 *μ*M), and the number of LDs after 1–24 h of incubation was determined. As shown in Figure 2(b), MT-II caused a significant increase (*P* < 0.05) in the numbers of LDs after 1–24 h incubation compared with control cells. The highest number of LDs was detected after 24 h incubation. As illustrated in Figure 2(c), control macrophages stained with OsO_4_ showed very few osmiophilic inclusions in the cytoplasm. In contrast, MT-II-stimulated macrophages exhibited a cytoplasm packed with the osmiophilic organelles, which can be seen as dark punctate structures in Figures 2(d), 2(e), and 2(f). 

### 3.3. Ultrastructural Analysis of LDs Induced by MT-II

**Figure 3:**
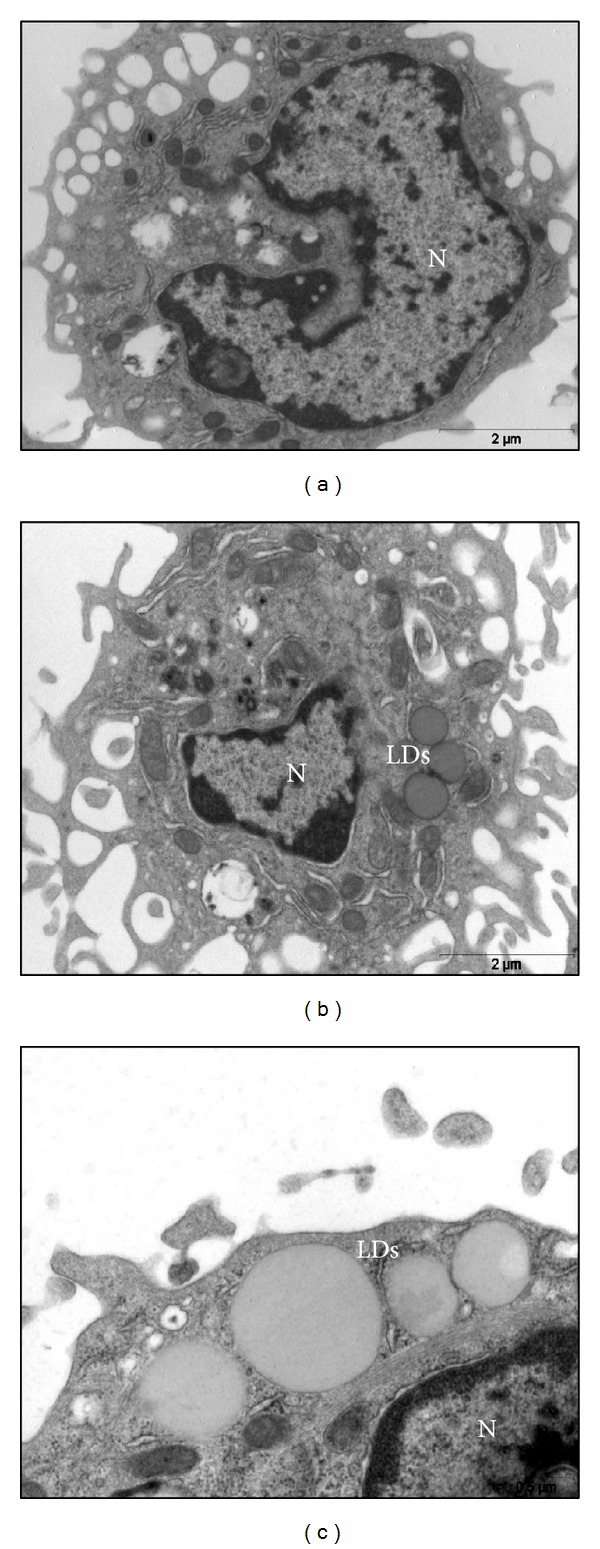
MT-II-induced LDs are morphologically distinct cytoplasmic sites. (a) Control macrophages with typical morphology. (b) and (c) Macrophages incubated with MT-II (0.8 *μ*M) for 1 h showing light and large cytoplasmic lipid droplets (LDs). Also, an enlarged ER can be observed in MT-II-stimulated cells. N: nucleus.

To further investigate the stimulatory effect of MT-II leading to LDs formation in macrophages, ultrastructural analysis of LDs was performed using a standardized procedure for TEM. As seen in Figure 3(a) control macrophages showed small, non-membrane-bound, light cytoplasmic LDs. After 1 h incubation, MT-II-stimulated macrophages showed light cytoplasmatic LDs that were present in markedly greater numbers than in the control cells but morphologically similar to LDs in these cells (Figure 3(b)). Also, an enlarged ER was observed in MT-II-stimulated cells in incubation period tested as showed in Figure 3(c). 

### 3.4. Effects of Peptides Corresponding to Selected Regions of MT-II Molecule on LD Formation

**Figure 4:**
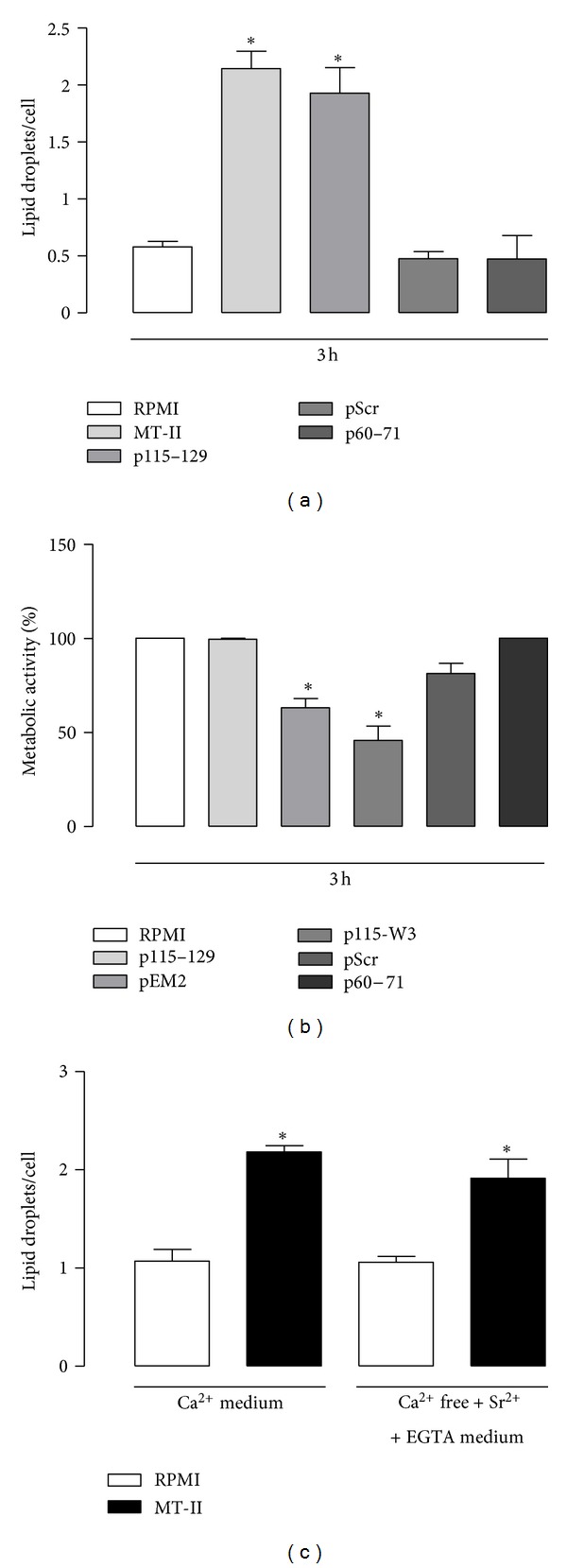
Effects of peptides corresponding to selected regions of MT-II molecule on LD formation. (a) Peritoneal macrophages were incubated with p115-129 (250 *μ*g/mL) or pScr (250 *μ*g/mL) or p60–71 (250 *μ*g/mL) or RPMI (control) for 3 h. LDs were quantified using light microscopy after osmium staining; (b) cells were incubated with p115–129 (250 *μ*g/mL) or pScr (250 *μ*g/mL) or p60–71 (250 *μ*g/mL) or PEM2 (150 *μ*g/mL) or p115–W3 (150 *μ*g/mL) or RPMI (control) for 3 h, after which cytotoxicity was assessed by the tetrazolium-based (MTT) colorimetric assay; (c) effect of MT-II (0.8 *μ*M) on formation of LDs in macrophages in a Ca^2+^ containing medium or Ca^2+^-free, EGTA (200 *μ*M)-Sr^2+^-containing medium. LDs were quantified using light microscopy after osmium staining. Each bar represents the mean ± S.E.M. of the number of LDs/cell in 50 counted cells. Values represent means ± S.E.M. for three to five animals. *P < 0.05 compared with control cells.

The effects of synthetic peptides derived from distinct regions of MT-II protein on LDs formation were investigated in macrophages. Experiments were carried out with macrophages stimulated with peptides corresponding to distinct regions of MT-II molecule for 3 h and then treated as necessary for lipid fixation and stained with OsO_4_.  Figure 4(a) demonstrates that incubation of macrophages with the C-terminal peptide p115-129 induced a significant increase (*P* < 0.05) in the number of LDs after 3 h of incubation in comparison with nonstimulated control cells. The effect induced by this C-terminal peptide did not differ from that observed in cells stimulated with MT-II native protein for 3 h, which caused a significant increase in the number of LDs in comparison with control cells. On the other hand, neither a scrambled version of the residue sequence used as a control, p-Scr, nor the peptide comprising amino acid residues 60–71 of central region of MT-II modified the basal numbers of LDs after 3 h of incubation as compared with RPMI-treated control cells. As a positive control macrophages were incubated with MT-II native protein for 3 h. In this case, a significant increase in LD number was detected in comparison with RPMI-treated control cells. All peptides tested were used in a concentration (250 *μ*g/mL) previously demonstrated in literature as effective to induce biologic effects (32), but without toxic effect on the viability of macrophages after 3 h of exposure.  Figure 4(b) demonstrates that incubation of cells with peptides p115–129, pScr, and p60–71 at a concentration of 250 *μ*g/mL did not affect macrophages viability, whereas incubation of cells with peptides pEM2 (150 *μ*g/mL) and p115-W3 (150 *μ*g/mL) significantly (*P* < 0.05) reduced the viability of macrophages making these peptides unsuitable for the present study. Although MT-II is recognized as a sPLA_2_ devoid of catalytic activity, we investigated whether a possible residual enzyme activity of MT-II would lead to formation of LDs in macrophages. As shown in Figure 4(c) incubation of macrophages with MT-II (0.8 *μ*M) for 1 h in the presence of a Ca^2+^-containing medium induced a significant increase (*P* < 0.05) in the number of LDs. This MT-II-induced effect was not modified in the presence of Ca^2+^-free, EGTA- and Sr_2_^+^-containing medium, with a significant increase of LD numbers observed in comparison with respective control group. 

### 3.5. LD Formation Triggered by MT-II Is Dependent on Distinct Signaling Pathways

**Figure 5:**
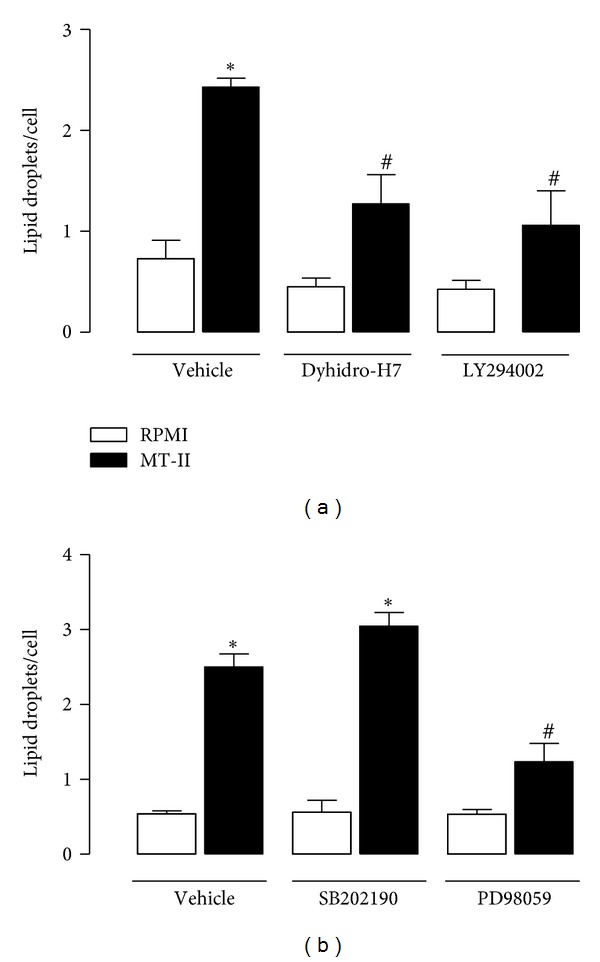
Signaling pathways involved in MT-II-induced LD formation. Peritoneal macrophages were incubated with one of the following: (a) PKC inhibitor H7 Dihidro (6 *μ*M) for 1 h or the PI3 K inhibitor LY294002 (1 *μ*M) for 1 h; (b) the p38MAPK or ERK1/2 inhibitors SB202190 (1 *μ*M) or PD98059 (25 *μ*M) for 1 h before stimulation with MT-II (0.8 *μ*M) for 1 h. LDs were counted using light microscopy after osmium staining. Each bar represents the mean ± SEM of the number of LDs/cell in 50 counted cells. Values represent means ± SEM from three to five animals. *P < 0.05 compared with control cells; ^#^ *P* < 0.05 compared with MT-II-stimulated cells.

 To assess the role of kinases in the described actions of MT-II, we determined the effects of the specific inhibitors of p38, PI3 K, PKC, and ERK1/2 (SB202190, LY294002, H7-Dihydro, and PD98059, resp.) on MT-II-induced LDs in macrophages. As seen in Figure 5(a), the PI3 K and PKC inhibitors abolished the LDs formation in MT-II-stimulated macrophages compared with vehicle-treated macrophages stimulated with MT-II. The ERK1/2 inhibitor, in turn, caused 49% reduction in the number of LDs in MT-II-stimulated macrophages when compared with vehicle-treated macrophages stimulated with MT-II (Figure 5(b)). In contrast, the preincubation of macrophages with p38MAPK inhibitor did not change the number of LDs induced by MT-II, in comparison to cells stimulated with MT-II only (Figure 5(b)).

### 3.6. MT-II Upregulates PLIN2 Protein Expression in MT-II-Stimulated Macrophages

**Figure 6:**
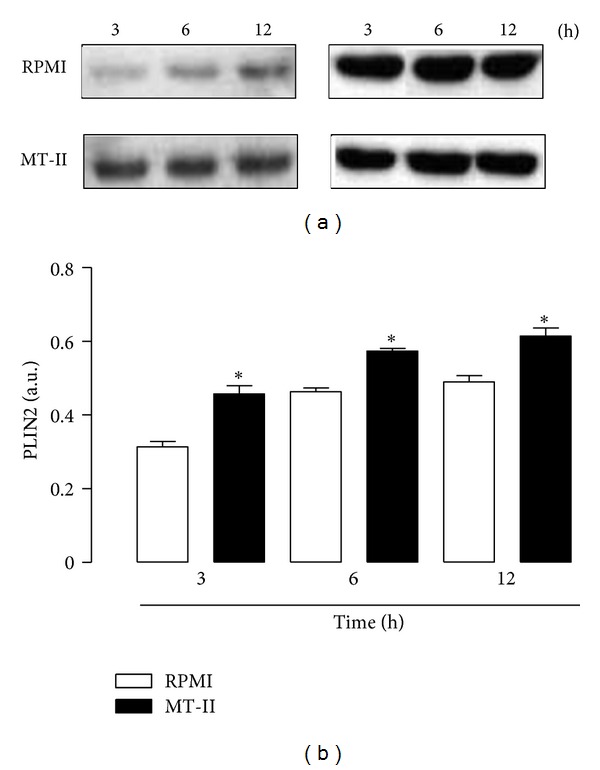
MT-II induces upregulation of PLIN2 expression in macrophages. Peritoneal macrophages were incubated with MT-II (0.8 *μ*M) or RPMI (Control) for 3, 6, and 12 h. (a) Western blotting of PLIN2 and *β*-actin (loading control) in macrophage extracts. (b) Densitometric analysis of the band intensities of immunoreactive PLIN2. The densities (in arbitrary units) were normalized with those of *β*-actin. Results are expressed as mean ± S.E.M. from three experiments. *P < 0.05 compared with controls.

PLIN2 expression can be induced by a variety of inflammatory cells and has been associated with increased numbers of LDs [26, 27]. Therefore, we investigated whether MT-II induces expression of this LD structural protein. Levels of PLIN2 protein expression were analyzed by western blotting in cells incubated and not incubated with MT-II for selected time periods. This analysis revealed increased expression of PLIN2 protein in cells stimulated with MT-II as early as 3 h of incubation, which was sustained up to 12 h. PLIN2 was minimally expressed or absent in control nonstimulated macrophages (Figures 6(a) and 6(b)). 

### 3.7. PLIN2 Colocalizes to LDs in MT-II-Stimulated Macrophages

**Figure 7:**
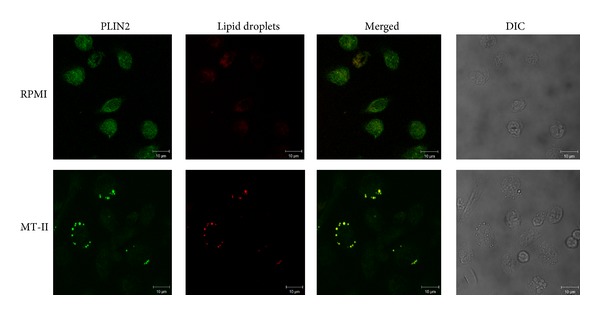
PLIN2 and LD colocalize in macrophages stimulated by MT-II. Macrophages incubated with RPMI (control) or MT-II (0.8 *μ*M) for 3 h were labeled for LDs (fluorescent Nile Red) and for PLIN2 (FITC-conjugated immunocomplex). Merged image shows colocalization of PLIN2 to LDs. Cell nuclei are observed by DIC. The pictures are representative of three independent experiments.

To better understand the stimulatory effect of MT-II on macrophages that leads to LD formation, cells exposed to MT-II were immunostained with specific antibodies that recognize PLIN2 or neutral lipids from the LD core. As illustrated in Figure 7 macrophages stimulated with MT-II (0.8 *μ*M) for 3 h exhibited strong fluorescent staining (green) for PLIN2, with a punctate cytoplasmic pattern, which was absent in the nonstimulated control cells. Fluorescent Nile Red-labeled LDs were also visualized 3 h after MT-II-induced stimulation and were virtually absent in nonstimulated control macrophages. After stimulation with MT-II, cytoplasmic-stained PLIN2 matched perfectly with Nile Red-marked, neutral lipid inclusions. As expected, no significant staining was detected in control macrophages. 

### 3.8. Involvement of Intracellular PLA2s in MT-II-Induced LD Formation in Macrophages

**Figure 8:**
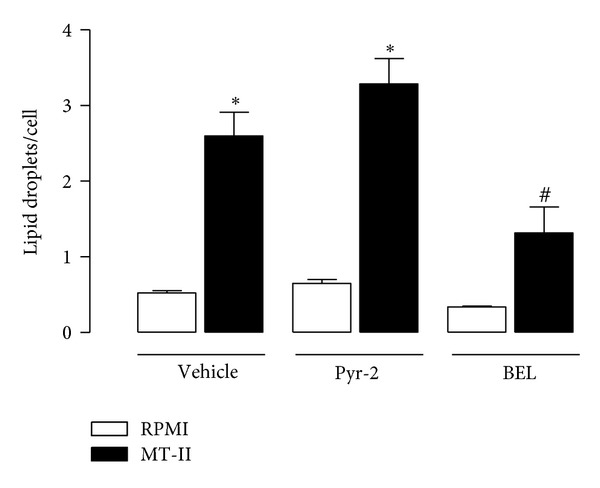
Effects of inhibitors of c cPLA_2_ and iPLA_2_ on MT-II induced LDs formation. Peritoneal macrophages were incubated with Pyr-2 (1 *μ*M) or BEL (2 *μ*M) compounds for 30 min and then with MT-II (0.8 *μ*M) for 3 h. LDs were quantified using light microscopy after osmium staining. Each bar represents the mean ± sem LDs/cell in 50 counted cells. Values represent means ± SEM from 3–5 animals. *P < 0.05 compared with control group; ^#^ *P* < 0.05 compared with MT-II-stimulated cells.

Since a cross-talk between sPLA_2_ and intracellular PLA_2_s for production of prostaglandins has been described, we examined the effects of selective inhibitors of cPLA_2 _(Pyr-2) or iPLA_2_ (BEL) on MT-II induced formation of LDs. As shown in Figure 8, treatment of macrophages with BEL, but not with Pyr-2 compound caused 51% reduction in the number of LDs in MT-II-stimulated macrophages compared with vehicle-treated cells stimulated with MT-II. These results indicate that iPLA_2_, but not cPLA_2_ is involved in MT-II-induced LD biogenesis.

### 3.9. PGE_2_ Colocalizes within LD Induced by MT-II-Stimulated Macrophages

**Figure 9:**
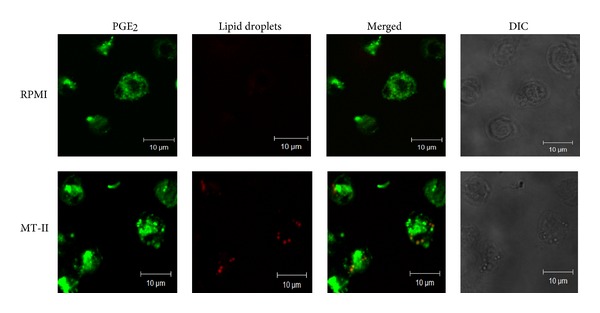
Cytoplasmic lipid droplets compartmentalize PGE_2_. Macrophages incubated with RPMI (control) or MT-II (0.8 *μ*M) for 3 h were labeled for LBs (fluorescent Nile Red) and for PGE_2_ (Cayman Chemical). Merged image shows colocalization of PGE_2_ to LDs. Cell nuclei are observed by DIC. The pictures are representative of three independent experiments.

Under inflammatory conditions, lipid mediators are mainly produced within LBs, which compartmentalize both substrate and the enzymatic machinery required for eicosanoid production [28]. Considering that PGE_2_ is the major prostaglandin produced in macrophages, we evaluated the subcellular localization of PGE_2_ within MT-II-stimulated macrophages. As illustrated in Figure 9 immunofluorescence microscopy revealed that macrophages stimulated with MT-II (0.8 *μ*M) for 3 h exhibited strong fluorescent staining (green) for PGE_2_, with a punctate cytoplasmic pattern, which was diffuse in the nonstimulated control cells. Fluorescent Nile Red-labeled LBs were also visualized 3 h after MT-II-induced stimulation and were virtually absent in nonstimulated control macrophages. Overlapping images show that after stimulation with MT-II, cytoplasmic-stained PGE_2_ matched perfectly with Nile Red-marked, neutral lipid inclusions. As expected, no significant staining was detected in control macrophages. 

## 4. Discussion

Besides myotoxic activity, MT-II, a Lys49PLA_2_ homologue, has been shown to activate some cellular processes in macrophages at noncytotoxic concentrations [4, 5], and these effects may contribute to the overall tissue alterations caused by this toxin. Upon inflammatory conditions, macrophages show increased numbers of cytoplasmic LDs, which have been implicated as key organelles involved in immunity and inflammation.

In this study, we showed that MT-II, a catalytically inactive PLA_2_ homologue, was able to directly induce an increase in the numbers of LDs in isolated murine macrophages. This phenomenon was time dependent and had a very fast onset and persisted up to 24 h after stimulation. Within 1 h of MT-II stimulus the presence of weakly osmiophilic LDs, in close association with organelles such as endoplasmic reticulum, were evidenced by the ultrastructural analysis. According to the current model of LD biogenesis, these organelles arise from endoplasmic reticulum, where the enzymes that synthesize lipids reside [15, 29]. Therefore, endoplasmic reticulum may play a role in LDs biogenesis induced by MT-II.

Because LDs have been associated to regulated inflammatory mediator synthesis with roles in inflammatory and infectious conditions, and macrophages are central elements in the innate immune response it is plausible to consider that biogenesis of LDs induced by MT-II demonstrated herein represents an important mechanism by which this PLA_2_ homologue displays an inflammatory response and leads to production and release of inflammatory mediators. Moreover, considering that basic PLA_2_s comprise around 30% of *B. asper* venom [30], the fact that MT-II elicited a key inflammatory event in macrophages clearly indicates that this sPLA_2_ homologue contributes to the local inflammatory response triggered by the whole venom. 

In addition, our data demonstrated that the absence of Ca^2+^ and presence of Sr^2+^ in culture medium did not alter LDs formation induced by MT-II, confirming that the catalytic activity is not an essential requirement to enhancement of LDs biogenesis by MT-II. A number of experimental evidences suggested that a stretch of residues, located at the C-terminus of the MT-II protein molecule, and involving cationic and hydrophobic amino acids are responsible for myotoxic and cytotoxic effects of this [9, 31, 32] and other Lys-49sPLA_2_ homologues [31, 33]. Based on these and other studies, a model of Lys49PLA_2_s-membrane interaction was proposed by Lomonte et al. [9] in which the action of Lys49PLA_2_s is based on the interaction of the C-terminal positive residues with membrane anionic phospholipids. So far, in the present study we found that the synthetic peptides 115–129 corresponding to MT-II C-terminus peptide induced LD formation in macrophages similarly to the parent protein. This finding indicates for the first time the specific region of MT-II molecule responsible for activation of macrophages, and gives support to the notion that the effects of MT-II in leukocytes are not related to the PLA_2_ enzymatic activity. The membrane target(s) and the mechanisms by which this C-terminal peptide triggers macrophages activation to form LDs were not addressed in this study, although perturbation of the membrane phospholipid bilayer is likely to be involved.

Perilipin 2 is a protein ubiquitously expressed in a number of cell types, including macrophages, as a major component of intracellular LDs [34, 35]. It has fatty acid-binding properties, contributes to cytoplasmic trafficking of newly synthesized lipids, and plays an important role in assembly of LDs as well as in foam cell formation [35–37]. PLIN2 expression can be induced by a variety of inflammatory stimuli and has been associated with increased numbers of LD [26, 27]. Consistent with its properties, PLIN2 has been considered as a marker of LDs assembly and lipid loading in inflammatory cells, such as macrophages. Accordingly, our results showed that PLIN2 protein expression is upregulated by MT-II, given support to data demonstrating LD formation upon stimulus by this PLA_2_ homologue. Furthermore, PLIN2 clustering co-localized to LDs was seen indicating that MT-II is also able to recruit this protein from its constitutive pools into LDs, and suggesting a role for PLIN2 as a nucleation site for the assembly of lipids to form new LDs under the stimulus of this Lys49PLA_2_. 

LD biogenesis in leukocytes is a highly regulated process. Studies of the intracellular signaling pathways committed to this process in leukocytes have revealed that distinct pathways can trigger LD biogenesis in a stimulus-dependent manner [38]. To better understand the stimulatory effects of MT-II on LD formation, we herein used pharmacological approaches to identify the critical downstream signaling proteins involved in LD formation induced by this PLA_2_ homologue and focused on major downstream signaling molecules that have previously been shown to participate in LD biogenesis which follows inflammatory stimuli, such as PKC [39, 40], PI3 K [16, 26] and MAPKs (p38^MAPK^ and ERK1/2) [16, 41]. As a marked LD formation was observed after 3 h of incubation, the effects of pharmacological compounds were evaluated at this time interval. We found that MT-II-induced LD formation is regulated by specific signaling pathways and that PKC, PI3 K, ERK1/2, but not p38^MAPK^ are involved in the formation of LD induced by this PLA_2_ homologue.

Our finding that macrophage activation by MT-II to form LDs is largely dependent on the PKC agrees with previous reports that PKC activation is implicated in LD formation induced by cys-fatty acid and PAF in leukocytes [39, 42]. Considering that activation of PKC has been associated with increased expression of PLIN2 in macrophages [43], it is possible to suggest that in the present experimental conditions, PKC signaling pathway is important to MT-II-induced up-regulation of PLIN2, and thus to the increased formation of LDs. Moreover, our observation that LDs formation induced by MT-II requires activation of the PI3 K pathway is in line with reports of participation of this signaling protein in processes related to lipid accumulation [26, 44, 45] and in the regulation of PLIN2 which has been largely associated to lipid accumulation into LDs, and to atherosclerosis [26, 45]. Furthermore, our results implicating ERK1/2 signaling in the MT-II effect that leads to LD formation in macrophages are consistent with previous studies demonstrating the involvement of ERK1/2 in LDs biogenesis induced by cytokines and saturated fatty acids in macrophages [16, 46]. Moreover, evidences of the involvement of ERK1/2 in regulation of PLIN2 expression and the growth of LDs [41] give support to our findings of increased protein expression of PLIN2 seen under MT-II stimulus, and is in line with the involvement of ERK1/2 in biogenesis of LDs induced by this PLA_2_ homologue. Conversely, the specific inhibitor of p38^MAPK^ failed to inhibit MT-II-induced LD formation, implying that this MAPK element does not contribute to this MT-II-induced effect. Taken together, the above results evidenced that MT-II-induced LD formation is a regulated process associated to activation of selected downstream signaling pathways in macrophages. Of note, despite the lack of enzyme activity, MT-II triggers signaling pathways almost similar to those that signal increased formation of LDs induced by the catalytically active sPLA_2_ MT-III in macrophages [22], thus providing an additional evidence of functional similarities between these two venom sPLA_2 _variants.

It has been demonstrated that LDs are involved in production of inflammatory mediators [28] and to act as platforms for enhanced PGE_2_ synthesis during infection conditions [47, 48]. Moreover, a number of enzymes and signaling proteins were shown to be associated with LDs, including the prostaglandin-forming enzymes named cyclooxygenases [47]. Our findings that MT-II caused an increase of PGE_2_ intracellular pools, which colocalized to LDs in macrophages represent the first evidence that a sPLA_2_ homologue is able to induce synthesis and compartmentalization of a lipid mediator in LDs. These findings suggest that macrophage LD constitutes a relevant site for the synthesis and accumulation of eicosanoids under MT-II stimuli and may represent a rapid and alternative mechanism for PGE_2_ production by which macrophages react to activation by this sPLA_2_ homologue. Moreover, given the importance of PGE_2_ in several inflammatory settings, due to its hyperalgesic and edematogenic properties [49, 50], it is reasonable to suggest that LD-derived PGE_2_ may have implications for the inflammatory effects of MT-II. This hypothesis is in line with the view that LDs are dynamic organelles integrating lipid metabolism, inflammatory mediator production, membrane trafficking, and intracellular signaling [27, 47, 51].

A number of studies have demonstrated that secreted PLA_2_s crosstalk with the intracellular PLA_2_s (cPLA_2_ and iPLA_2_) to produce arachidonic acid- (AA-) derived inflammatory mediators, such as prostaglandins, in several pathophysiological conditions [52, 53]. cPLA_2_ is recognized as a key regulator of stimulus-coupled cellular AA release [6, 54]. The iPLA_2_ in turn has no substrate specificity for the fatty acid residue at *sn-2* position, playing a minor role in eicosanoid synthesis. This intracellular enzyme, however, has a role in membrane phospholipid remodeling through deacylation/reacylation reactions [55]. In this context, cPLA_2_ and iPLA_2_ were demonstrated to be involved in LD biogenesis induced by stress in CHO-K1 cells [56] and by the Asp49PLA_2_ MT-III in macrophages [22]. Taking the above information into account we investigated the participation of both intracellular PLA_2_ isoforms in MT-II-induced LD formation. We found that treatment of macrophages with, compound Pyr-2, a specific inhibitor of cPLA_2_ failed to inhibit MT-II-induced effect, indicating that cPLA_2_ is not required for LD formation under MT-II stimulus. This finding is in accordance with our observation that p38^MAPK^ is not involved in LD formation induced by MT-II, as upstream p38^MAPK^ is critical for phosphorylation and activation of cPLA_2_ [57]. In contrast, inhibition of iPLA_2_ by BEL compound significantly reduced LD formation, thus implying iPLA_2_ in the mechanisms involved in MT-II-induced LD formation. This finding is supported by recent studies demonstrating participation of iPLA_2_s in the metabolism of fatty acids and triacylglycerol formation, which are involved in LD formation [56]. We believe that our results are the first demonstration that a sPLA_2_ devoid of catalytic activity recruits an intracellular PLA_2_ (iPLA_2_) to induce a cellular event, such as LD formation in macrophages. However, the cellular steps involved in such a protein crosstalk were not addressed in the present study and deserve further studies. 

## 5. Conclusions

Taken together, our data show that the venom group IIA sPLA_2_ homologue MT-II directly activates murine macrophages to form LDs by a mechanism independent on enzymatic activity. This effect is related to the C-terminal loop of the MT-II molecule since a synthetic peptide corresponding to region 115–129-induced LD formation similarly to MT-II. Moreover, MT-II-induced LD formation is related to increased expression and recruitment of PLIN2 from its constitutive pools and regulated by distinct signaling pathways that include PKC, PI3 K, ERK1/2, and iPLA_2_. In addition, MT-II induced synthesis and compartmentalization of PGE_2_ within LDs. Therefore, LDs may represent and important platform for the synthesis and accumulation of lipid mediators under MT-II stimulus that takes place in the mechanisms whereby this Lys49PLA_2_ triggers inflammation.

Finally, considering that catalytically inactive PLA_2_s have been also described in mammalian tissues under normal and pathological conditions [58, 59], this study may shed light on the possible activities of similar proteins on a more general scope, providing insights into the possible roles of human catalytically inactive PLA_2_ homologues in inflammatory conditions as it has been demonstrated for the snake venom Lys49PLA_2_.
